# Effects of Manipulating Circulating Bile Acid Concentrations on Postprandial GLP-1 Secretion and Glucose Metabolism After Roux-en-Y Gastric Bypass

**DOI:** 10.3389/fendo.2021.681116

**Published:** 2021-05-14

**Authors:** Isabella Jonsson, Kirstine N. Bojsen-Møller, Viggo B. Kristiansen, Simon Veedfald, Nicolai J. Wewer Albrechtsen, Trine R. Clausen, Rune E. Kuhre, Jens F. Rehfeld, Jens J. Holst, Sten Madsbad, Maria S. Svane

**Affiliations:** ^1^ Department of Endocrinology, Hvidovre Hospital, Hvidovre, Denmark; ^2^ Novo Nordisk Foundation Center for Basic Metabolic Research and Department of Biomedical Sciences, University of Copenhagen, Copenhagen, Denmark; ^3^ Department of Surgical Gastroenterology, Hvidovre Hospital, Hvidovre, Denmark; ^4^ Department of Clinical Biochemistry Rigshospitalet, Copenhagen, Denmark; ^5^ Novo Nordisk Foundation Center for Protein Research, University of Copenhagen, Copenhagen, Denmark; ^6^ Research and Development, Novo Nordisk A/S, Måløv, Denmark

**Keywords:** bile acids, colesevelam, RYGB, Roux-en-Y gastric bypass, glucagon-like peptide 1

## Abstract

**Background:**

Altered bile acid (BA) turnover has been suggested to be involved in the improved glucose regulation after Roux-en-Y gastric bypass (RYGB), possibly *via* stimulation of GLP-1 secretion. We investigated the role of exogenous as well as endogenous BAs for GLP-1 secretion after RYGB by administering chenodeoxycholic acid (CDCA) and the BA sequestrant colesevelam (COL) both in the presence and the absence of a meal stimulus.

**Methods:**

Two single-blinded randomized cross-over studies were performed. In study 1, eight RYGB operated participants ingested 200 ml water with 1) CDCA 1.25 g or 2) CDCA 1.25 g + colesevelam 3.75 g on separate days. In study 2, twelve RYGB participants ingested on separate days a mixed meal with addition of 1) CDCA 1.25 g, 2) COL 3.75 g or 3) COL 3.75 g × 2, or 4) no additions.

**Results:**

In study 1, oral intake of CDCA increased circulating BAs, GLP-1, C-peptide, glucagon, and neurotensin. Addition of colesevelam reduced all responses. In study 2, addition of CDCA enhanced meal-induced increases in plasma GLP-1, glucagon and FGF-19 and lowered plasma glucose and C-peptide concentrations, while adding colesevelam lowered circulating BAs but did not affect meal-induced changes in plasma glucose or measured gastrointestinal hormones.

**Conclusion:**

In RYGB-operated persons, exogenous CDCA enhanced meal-stimulated GLP-1 and glucagon secretion but not insulin secretion, while the BA sequestrant colesevelam decreased CDCA-stimulated GLP-1 secretion but did not affect meal-stimulated GLP-1, C-peptide or glucagon secretion, or glucose tolerance. These findings suggest a limited role for endogenous bile acids in the acute regulation of postprandial gut hormone secretion or glucose metabolism after RYGB.

## Introduction

Roux-en-Y gastric bypass (RYGB) is the most effective treatment of both obesity and type 2 diabetes (1, 2). Interestingly, glucose metabolism is improved in patients with type 2 diabetes within days after surgery indicating causal mechanisms other than weight loss (3, 4). Several factors seem to be involved including an accelerated transit of nutrients to the small intestine and augmented absorption, which leads to altered gut hormone secretion and among these a greatly enhanced (up to 10-fold) secretion of glucagon-like peptide-1 (GLP-1) (5, 6). GLP-1 is an insulinotropic and appetite inhibiting hormone, and in studies involving the GLP-1 receptor antagonist, Exendin 9-39, the GLP-1 response has been shown to be important for the reduction in food intake and improved beta-cell function observed after RYGB (7–9).

The mechanisms driving the increased secretion of GLP-1 are not fully clarified, but bile acids have been suggested to play a role. Apart from their role in lipid digestion and absorption, bile acids are signal molecules that stimulate GLP-1 secretion through activation of the G-protein coupled receptor TGR-5 (10), as demonstrated both *in vivo* (11) and *in vitro* (12–17). Moreover, activation of the bile-acid sensitive nuclear receptor FXR has also been suggested to modulate GLP-1 secretion (18, 19). After RYGB, increased concentrations of plasma bile acids are positively correlated to the increase in GLP-1 concentrations (20, 21) and improved glucose tolerance (22, 23) months to years after RYGB. These observations led to the hypothesis that bile acids are involved in the metabolic improvements after RYGB. However, there is a dissociation in the timing of the postoperative changes since total plasma bile acid concentrations are unchanged in the early postoperative period (21, 24), whereas postprandial GLP-1 secretion is enhanced immediately after surgery, concomitantly with increased insulin secretion and improved glucose tolerance (25, 26). Thus, the role of bile acids for the exaggerated postprandial GLP-1 secretion and glucose metabolism after RYGB remains unclear.

We have previously demonstrated that administration of the unconjugated primary bile acid chenodeoxycholic acid (CDCA) [a potent TGR5 and FXR agonist (27, 28)] to RYGB operated subjects stimulates both GLP-1, PYY, neurotensin, glucagon, and insulin secretion even in the absence of nutrient ingestion.

The primary aim of the current study was to expand on this observation of a link between bile acids and GLP-1 secretion by examining the effect of CDCA in a different cohort and during meal-ingestion and, more importantly, to investigate the contribution of endogenous bile acids for meal-stimulated GLP-1 secretion. The latter was done by inhibiting bile acid reabsorption through administration of the bile acid sequestrant colesevelam (COL), which efficiently blocks reabsorption by cross-linking of bile acids in the intestinal lumen leading to increased fecal bile acid excretion (29, 30). As acute bile acid-stimulated GLP-1 secretion has been demonstrated to be mediated by activation of basolaterally located TGR5 receptors on the L-cell (27, 31), blocking reabsorption would be expected to reduce GLP-1 secretion. The ability of COL to attenuate bile acid absorption and decrease GLP-1 secretion was tested by administration of exogenous CDCA with and without COL in the absence of nutrients in a sub-study (study 1) before testing the postprandial responses (study 2). In addition, we investigated the modulatory effects of exogenous (CDCA) and endogenous bile acids (by COL administration) on meal-induced plasma concentrations of glucose, including the risk of hypoglycemia, and other glucoregulatory hormones including insulin, glucagon, CCK, neurotensin and FGF-19. Apart from in the N-cell, neurotensin is co-expressed with GLP-1 in some L-cells (32) and its secretion, like GLP-1, depends on intestinal absorption and receptor activation, which might be modulated by CDCA and COL (27, 32).

## Methods

### Participants

For study 1, eight participants (four women and four men), and for study 2 twelve participants (six women and six men) were recruited from the bariatric surgery program at Hvidovre Hospital (**Tables 1** and **2**).

**Table 1:** Study participants from study 1 (n = 8). Data are presented as mean ± SEM.

Age (years)	49.0 ± 2.6
Time since RYGB (months)	60.1 ± 5.8
Weight loss (kg)	22.7 ± 4.6
Weight (kg)	106 ± 9.9
Body mass index (kg/m^2^)	35.1 ± 2.1
Fasting plasma glucose (mmol/L)	5.3 ± 0.2
HbA1c (mmol/mol)	38.8 ± 2.1

**Table 2:** Study participants from study 2 (n = 12). Data are presented as mean ± SEM.

Age (years)	43.1 ± 2.6
Time since RYGB (months)	51.5 ± 5.4
Weight loss (kg)	33.8 ± 5.1
Weight (kg)	98.4 ± 6.8
Body mass index (kg/m^2^)	31.5 ± 1.9
Fasting plasma glucose (mmol/L)	5.3 ± 0.1
HbA1c (mmol/mol)	36.5 ± 1.3

Inclusion criteria for both studies were uncomplicated RYGB >3 months prior to inclusion and only participants without diabetes were included (defined as fasting plasma glucose <7.0 mmol/L and HbA1c <48 mmol/mol before and after RYGB). Exclusion criteria were hemoglobin <6.5 mmol/L, cholecystectomy, dysregulated thyroid diseases (TSH outside reference range), use of anti-thyroid medication, renal insufficiency, prior pancreatitis, certain RYGB related complications (severe reactive hypoglycemia or dysphagia), pregnancy or lactation. All participants were weight stable at inclusion (defined as +/− 2 kg within the last month), and none had symptoms of postprandial hypoglycemia. Written informed consent was obtained from all participants before entering the study. The protocols were approved by the Regional Ethical Committee of the Capital Region in accordance with the Helsinki II declaration, by the Danish Data Protection Agency, and both studies were registered at www.clinicaltrial.org (NCT02952963 and NCT02876484).

### Study Design

In both studies, participants were instructed to refrain from strenuous physical activity and alcohol for three days and to fast overnight before all study days, which were separated by at least 48 h. On each study day participants were weighed, and a catheter was inserted into a forearm vein for blood sampling.

In study 1, participants were examined on two separate study days performed in randomized order with the following interventions: CDCA: Intake of 1.25 g chenodeoxycholic acid (Xenobilox capsules, Juers Pharma, Germany) suspended in 200 ml water, followed by 50 ml water; CDCA + COL: Intake of 1.25 g chenodeoxycholic acid and 3.75 g colesevelam suspended in 200 ml water followed by 50 ml water. After three basal blood samples (t = −10, −5, 0 min) CDCA or CDCA + COL was ingested evenly during a 5-min supervised period. Blood was sampled frequently for a total of 4 h (at *t* = 5, 10, 15, 20, 25, 30, 45, 60, 90, 120, 180, 240 min).

In study 2, participants were examined on four separate study days performed in a randomized order: 1. Meal: Mixed meal (1,523 kJ, 53E% carb, 33E% fat, 14E% protein) consisting of both solid and semisolid components: yoghurt, oatmeal, raisins, rye bread, margarine, cheese, and almonds) and with 1 g of paracetamol (PCM) (Pamol, Nycomed, Roskilde, Denmark) added to estimate pouch emptying, 2. Meal + CDCA: Mixed meal with 1.25 g chenodeoxycholic acid (Xenobilox capsules, Juers Pharma, Germany) mixed into the yoghurt, 3. Meal + COL Mixed meal with 3.75 g colesevelam suspended in 25 ml water and mixed into the yoghurt, 4. Meal + COL × 2: Mixed meal with 2 × 3.75 g colesevelam with the first dose of colesevelam given as capsules the night before the study day and the second dose given with the mixed meal as described for meal + COL.

At *t* = 0 the mixed meal was ingested during 20 supervised minutes, where participants were instructed to ingest the different components of the meal in the same order on every study day. PCM was added to the first bite of the meal. Blood was sampled as in study 1.

The doses of CDCA and COL reflected the clinically recommended daily doses of both drugs. We administered COL in two dosing regimens on separate experimental days, one involving a single dose immediately prior to meal intake and the other with an extra dose of COL given the night before the study day in addition to the dose with the mixed meal. This strategy was chosen because recent scintigraphic data suggests that bile acids in RYGB-operated subjects may be released independently of meal-ingestion and are present in the small intestine already in the fasting state (pre-meal) due to an altered bilio-enteric flow after RYGB, which may be mediated by enhanced passive re-uptake and decreased fecal bile acid loss (33, 34).

### Sample Collection and Laboratory Analyses

Blood was collected into a) prechilled EDTA-coated tubes containing in addition the dipeptidyl peptidase-4 substrate valine-pyrrolidide (final concentration 0.01 mmol/L; courtesy of Novo Nordisk) for analyses of plasma GLP-1, CCK, neurotensin, total bile acids (TBA) and fibroblast growth factor 19 (FGF-19) and b) clot activator tubes for analysis of serum C-peptide, insulin, and PCM (PCM, only in study 2).

EDTA tubes were centrifuged at 4°C (2,000 × *g* for 10 min) immediately after blood collection, and clot activator tubes after 30 min at room temperature for coagulation.

Plasma for analysis of plasma glucose was stored at 5°C before analysis at the end of each experimental day, using YSI model 2300 STAT plus (YSI, Yellow Spring, OH). Plasma and serum for all other analyses were stored at −20 and −80°C, respectively until batch analysis. Serum C-peptide concentrations were measured using an Immulite 2000 analyzer (Siemens Healthcare Diagnostics Inc., Tarrytown, NY, USA). Plasma samples were assayed for total GLP-1 and total neurotensin immunoreactivity using inhouse RIA using antiserum 89390 and 3D97, respectively, as reported previously (35, 36). CCK was measured by RIA using antiserum no. 92128, which binds all the bioactive forms of CCK in circulation (CCK-58, -33, -22, -8) with equal potency as reported previously (37). TBA concentrations were determined using an enzyme cycling based TBA Assay Kit from Diazyme (Cat No DZ042A-K, San Diego, CA, USA). FGF19 concentrations were determined using a commercial human FGF19 sandwich ELISA kit from BioVendor (Cat No RD191107200R, BioVendor, Brno, Czech Republic). Paracetamol was measured using a colorimetric assay (Roche Diagnostics GmbH, Mannheim, Germany).

### Statistical Analyses and Calculations

Basal concentrations were estimated as the mean of the three fasting samples. Total area-under-the-curve (AUC) was calculated using the trapezoidal rule and incremental AUC (iAUC) by subtracting baseline values. Positive incremental area-under-the-curve (piAUC) was calculated from postprandial concentrations above baseline concentrations. Beta-cell function was evaluated by two indices based on the results obtained from the mixed meal tests in study 2. Firstly, to estimate the initial beta-cell response during a meal, the insulinogenic index (IGI) was calculated from plasma C-peptide and plasma glucose: (C-peptide_t = 30_ – C-peptide_t = 0_)/(plasma glucose _t = 30_ – plasma glucose _t = 0_). Secondly, an index of beta-cell function covering the whole postprandial period was calculated as AUC_C-peptide_/AUC_glucose_ throughout the 240 min of the mixed meal test. Insulin resistance was calculated using the homeostasis model assessment of insulin resistance (HOMA-IR: insulin_fasting_ × glucose_fasting_/(22.5 × 6.945)). Rate of intestinal nutrient entry was evaluated by using time to peak of plasma paracetamol concentrations.

In study 1, effects of CDCA alone were evaluated as iAUC *vs*. 0 using paired t-tests, and differences between the two study days were analyzed using paired t-tests.

In study 2, the importance of exogenous and endogenous bile acids for outcome parameters was analyzed separately. Accordingly, the test days with meal + CDCA were compared with meal alone using paired t-tests, and the two days with COL administration were compared with meal in a one-way ANOVA with *post hoc* comparisons using Wald tests. Logarithmic transformations were used where appropriate. Data are expressed as mean ± SEM. P-values <0.05 were considered significant. Statistical analyses were performed in R version 3.5.1 (http://www.r-project.org/).

## Results

### Study 1

#### Total bile acids

**Figure 1:**
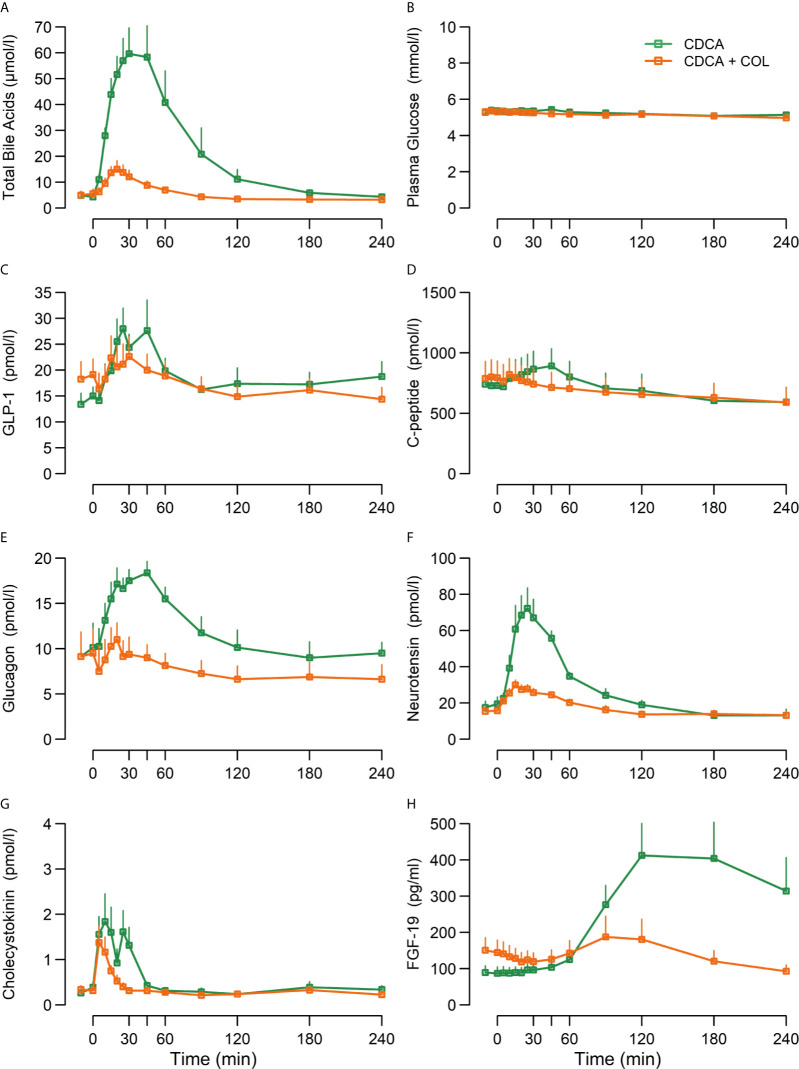
Concentrations of **(A)** total bile acids, **(B)** plasma glucose, **(C)** GLP-1, **(D)** C-peptide, **(E)** glucagon, **(F)** neurotensin, **(G)** cholecystokinin, and **(H)** FGF-19 following ingestion of chenodeoxycholic acid (CDCA) or CDCA in combination with the bile acid sequestrant colesevelam (COL) suspended in water (CDCA + COL), n = 8. Data are mean ± SEM.

TBAs increased markedly after CDCA administration, and increases were almost eliminated (~90%) when COL was co-administered (CDCA + COL) compared with CDCA alone (*p* < 0.01 for piAUC and peak values) (**Figure 1**, **Table 3**).

**Table 3:** Concentrations of glucometabolic- and gut hormones, VAS and blood pressure in response to CDCA administration with or without COL in study 1 (n = 8).

	CDCA	CDCA + COL	CDCA *vs.* COL
Basal GLP-1 (pmol/L)	14.2 ± 1.9	18.7 ± 3.1	*p* = 0.1
Peak GLP-1 (pmol/L)	34.8 ± 5.0	27.8 ± 4.2	*p* = 0.3
piAUC GLP-1 (pmol/L × min)	1213 ± 218	394 ± 209	*p < *0.01
Basal glucose (mmol/L)	5.4 ± 0.2	5.3 ± 0.2	*p* = 0.7
Peak glucose (mmol/L)	5.5 ± 0.1	5.4 ± 0.2	*p =* 0.1
piAUC glucose (mmol/L × min)	24.2 ± 6.4	42.9 ± 11	*p* = 0.2
Basal C-peptide (pmol/L)	732 ± 141	794 ± 141	*p* = 0.06
Peak C-peptide (pmol/L)	919 ± 142	855 ± 137	*p =* 0.1
piAUC C-peptide (pmol/L × min)	7667 ± 1542	555 ± 282	*p < *0.01
IGI_C-peptide_ (pmol/L/mmol/L)	582 ± 437	−1458 ± 1725	*p = 0.28*
Basal insulin (pmol/L)	48.2 ± 14	57.1 ± 16	*p* = 0.07
Basal glucagon (pmol/L)	9.6 ± 2.4	9.3 ± 2.7	*p = 0.86*
Peak glucagon (pmol/L)	20.0 ± 1.4	12.8 ± 1.9	*p < 0.01*
piAUC glucagon (pmol/L × min)	734 ± 265	253.4 ± 154	*p = 0.17*
HOMA-IR	1.7 ± 0.5	2.0 ± 0.6	*p* = 0.07
Basal CCK (pmol/L)	0.3 ± 0.07	0.3 ± 0.1	*p* = 0.9
Peak CCK (pmol/L)	2.5 ± 0.6	1.6 ± 0.3	*p* = 0.09
piAUC CCK (pmol/L × min)	52.5 ± 19	23.2 ± 3.9	*p* = 0.14
Basal NT (pmol/L)	18.5 ± 3.7	16.1 ± 3.0	*p = 0.4*
Peak NT (pmol/L)	83.3 ± 11	32.0 ± 2.3	*p < 0.01*
piAUC NT (pmol/L × min)	2,683 ± 441	1,114 ± 291	*p < 0.01*
Basal TBA (μmol/L)	4.6 ± 1.3	5.3 ± 1.6	*p =* 0.5
Peak TBA (μmol/L)	66.4 ± 11	15.8 ± 3.5	*p < *0.01
piAUC TBA (μmol/L × min)	3,916 ± 873	380 ± 86	*p < *0.01
Basal FGF-19 (pg/ml)	88.0 ± 18	147 ± 35	*p* = 0.2
Peak FGF-19 (pg/ml)	480 ± 109	198 ± 56	*p =* 0.01
piAUC FGF-19 (pg/ml × min)	47,978 ± 13,416	3,663 ± 2,018	*p =* 0.01

CDA, Chenodeoxycholic acid; COL, colesevelam; GLP-1, glucagon-like peptide-1; CCK, cholecystokininCCK, cholecystokinin; NT, neurotensin; TBA, total bile acids; FGF19, fibroblast growth factor 19. AUC, Area-under-the curve; piAUC, AUC above basal values. Data are presented as mean ± SEM.

#### GLP-1, Neurotensin, and CCK

CDCA clearly increased concentrations of GLP-1, NT, and CCK (iAUC *vs.* 0, p < 0.01) and administration of CDCA + COL decreased the responses of both GLP-1 (piAUC by 67%, p < 0.01) and neurotensin (piAUC by 58%, p < 0.01) compared with CDCA alone. Neither piAUC nor peak of CCK differed between study days, but addition of COL shortened the duration of the CCK response (**Figure 1**, **Table 3**).

#### Plasma Glucose, Serum C-Peptide, Glucagon and FGF-19

Plasma glucose was unaltered by CDCA, whereas C-peptide, glucagon, and FGF-19 increased significantly (iAUC *vs.* 0, p < 0.01). Neither peak nor piAUC of plasma glucose concentrations differed between study days, but concentrations of C-peptide were lower after CDCA + COL compared with CDCA alone (piAUC *p* < 0.01), (**Table 3**, **Figure 1**). For glucagon, peak concentrations decreased after CDCA + COL compared with CDCA alone (p < 0.01), and a numerical decrease was seen for piAUC (p = 0.17). Concentrations of FGF-19 decreased by >90% after CDCA + COL compared with CDCA (p = 0.01 for both piAUC and peak) (**Figure 1**, **Table 3**).

### Study 2

#### Total Bile Acids

**Figure 2:**
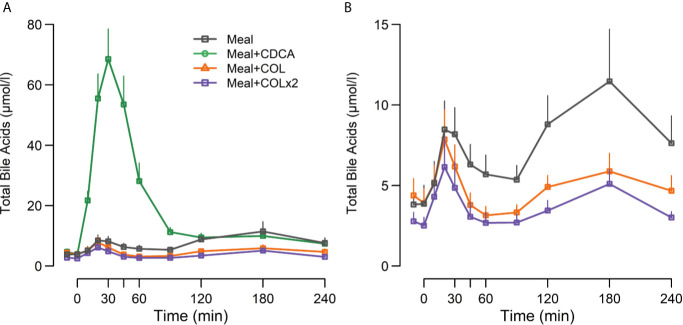
Plasma concentrations of **(A)** total bile acids and **(B)** total bile acids (without the CDCA concentration curve drawn) following ingestion of a mixed meal alone (Meal), mixed meal in combination with CDCA added to the meal (Meal + CDCA), mixed meal in combination with COL added to the meal (Meal + COL), or mixed meal in combination with COL administered both the night before the study day as tablets and added to the meal (Meal + COL × 2), n = 12. Data are mean ± SEM.

Plasma TBA concentrations increased markedly during meal + CDCA compared with meal alone (both peak and piAUC *p* < 0.01). On the contrary, a significant decrease of piAUC of TBA by ~68% was seen after both doses of COL (both *p* < 0.01) (**Figure 2**, **Table 4**).

**Table 4:** Concentrations of glucometabolic- and gut hormones, VAS and blood pressure in response to a mixed meal alone (Meal), meal with CDCA administration (Meal + CDCA), meal with single dosage of COL (Meal + COL) and meal with double dosage of COL (Meal + COL × 2) in study 2.

	Meal + CDCA	Meal + CDCA	Meal + COL	Meal + COLx2	t-test Meal *vs*. meal + CDCA	ANOVA (Meal, COL, COL × 2)	p Meal *vs*. COL	p Meal *vs*. COL × 2
	n = 12	n = 12	n = 12	*n = 9*
Basal GLP-1 (pmol/L)	14.0 ± 1.7	10.7 ± 1.5	13.0 ± 1.5	13.4 ± 2.4	p = 0.07	p = 0.93	–	–
Peak GLP-1 (pmol/L)	58.3 ± 6.1	65.3 ± 9.3	57.3 ± 4.6	52.4 ± 5.0	p = 0.3	p = 0.74	–	–
piAUC GLP-1 (pmol/L × min)	2867 ± 286	4362 ± 618	2703 ± 330	3328 ± 403	p = 0.04	p = 0.40	–	–
Basal Glucose (mmol/L)	5.3 ± 0.1	5.4 ± 0.1	5.3 ± 0.1	5.3 ± 0.1	p = 0.4	p = 0.99	–	–
Peak Glucose (mmol/L)	8.9 ± 0.3	8.3 ± 0.3	9.1 ± 0.3	9.2 ± 0.5	p = 0.03	p = 0.88	–	–
Nadir Glucose (mmol/L)	4.6 ± 0.1	4.7 ± 0.1	4.6 ± 0.1	4.5 ± 0.1	p = 0.49	p = 0.97		
piAUC Glucose (mmol/L × min)	154 ± 21	129 ± 15	153 ± 14	181.2 ± 24	p = 0.07	p = 0.69	–	–
Basal C-peptide(pmol/L)	714 ± 105	686.5 ± 111	676 ± 107	755 ± 124	p = 0.4	p = 0.88	–	–
Peak C-peptide (pmol/L)	3,558 ± 319	3,205 ± 321	3,097 ± 303	3,312 ± 381	p = 0.07	p = 0.58	–	–
piAUC C-peptide (nmol/L × min)	373 ± 41	346 ± 37	354 ± 42	390 ± 50	p = 0.04	p = 0.17	–	–
Basal glucagon (pmol/L)	8.8 ± 1.5	10.0 ± 1.5	9.0 ± 1.7	10.4 ± 1.5	p = 0.34	p = 0.76	–	–
Peak glucagon (pmol/L)	15.4 ± 1.6	19.4 ± 1.4	14.1 ± 1.4	15.6 ± 1.5	p = 0.004	p = 0.75	–	–
iAUC glucagon (pmol/L × min)	408 ± 183	615 ± 294	-69 ± 233	120 ± 221	p = 0.46	p = 0.27	–	–
Basal neurotensin (pmol/L)	19.6 ± 4.0	20.8 ± 2.5	23.1 ± 4.4	15.6 ± 3.5	p = 0.75	p = 0.47	–	–
Peak neurotensin (pmol/L)	173 ± 28	174 ± 21	133 ± 16	142 ± 18	p = 0.91	p = 0.40	–	–
piAUC neurotensin (pmol/L × min)	17,280 ± 2,242	16,069 ± 2,116	14,353 ± 1,630	18,033 ± 1829	p = 0.21	p = 0.38	–	–
Basal insulin (pmol/L)	48.5 ± 12	46.1 ± 11	43.4 ± 11	54.4 ± 16	p = 0.5	p = 0.84	–	–
HOMA-IR	1.7 ± 0.5	1.6 ± 0.4	1.5 ± 0.4	1.9 ± 0.6	p = 0.5	p = 0.87	–	–
Beta cell-index	288 ± 25	266 ± 25	272 ± 29	294 ± 20	p = 0.04	p = 0.85	–	–
IGI_C-peptide_	708 ± 67	673 ± 56	555 ± 63	598 ± 82	p = 0.48	p = 0.27	-	-
Basal CCK (pmol/L)	0.55 ± 0.1	0.59 ± 0.1	0.48 ± 0.1	0.52 ± 0.2	p = 0.71	p = 0.92	–	–
Peak CCK (pmol/L)	7.4 ± 1.4	6.0 ± 0.8	9.1 ± 1.7	8.2 ± 1.7	p = 0.1	p = 0.75	–	–
piAUC CCK (pmol/L × min)	405 ± 80	405 ± 64	567 ± 100	427 ± 78	p = 0.99	p = 0.36	–	–
Basal TBA (μmol/L)	3.9 ± 1.1	4.3 ± 0.9	4.2 ± 1.0	2.6 ± 0.7	p = 0.8	p = 0.57	–	–
Peak TBA (μmol/L)	13.7 ± 3.0	72 ± 11	9.9 ± 1.6	7.8 ± 2.2	p < 0.01	p = 0.38	–	–
piAUC TBA (μmol/L × min)	1,040 ± 204	3,642 ± 54	324 ± 85	329 ± 100	p < 0.01	p < 0.01	p < 0.01	p < 0.01
Basal FGF-19 (pg/ml)	185 ± 48	134 ± 27	236 ± 98	69 ± 14	p = 0.8	p = 0.27	-	-
Peak FGF-19 (pg/ml)	348 ± 70	632 ± 72	257 ± 81	136 ± 41	p < 0.01	p = 0.14	-	-
piAUC FGF-19 (pg/ml × min)	9,923 ± 3,642	48,905 ± 10,121	1,752 ± 766	5,520 ± 3,468	p < 0.01	p = 0.12	–	–
Time to peak, PCM (min)	16.3 ± 1.8	26.7 ± 9.0	18.3 ± 1.9	16.7 ± 2.9	p = 0.3	P = 0.74	–	–

CDA, Chenodeoxycholic acid; COL, colesevelam; COL × 2, colesevelam administered before and at the study day; GLP-1, glucagon-like peptide-1; CCK, cholecystokinin; NT, neurotensin; TBA, total bile acids; FGF19, fibroblast growth factor 19; AUC, Area-under-the curve; piAUC, piAUC above basal values; iAUC, iAUC with baseline subtracted values; tAUC_C-pep/_tAUC_PG+_, Beta cell-index. Data are presented as mean ± SEM.

#### GLP-1, Neurotensin, and CCK

**Figure 3:**
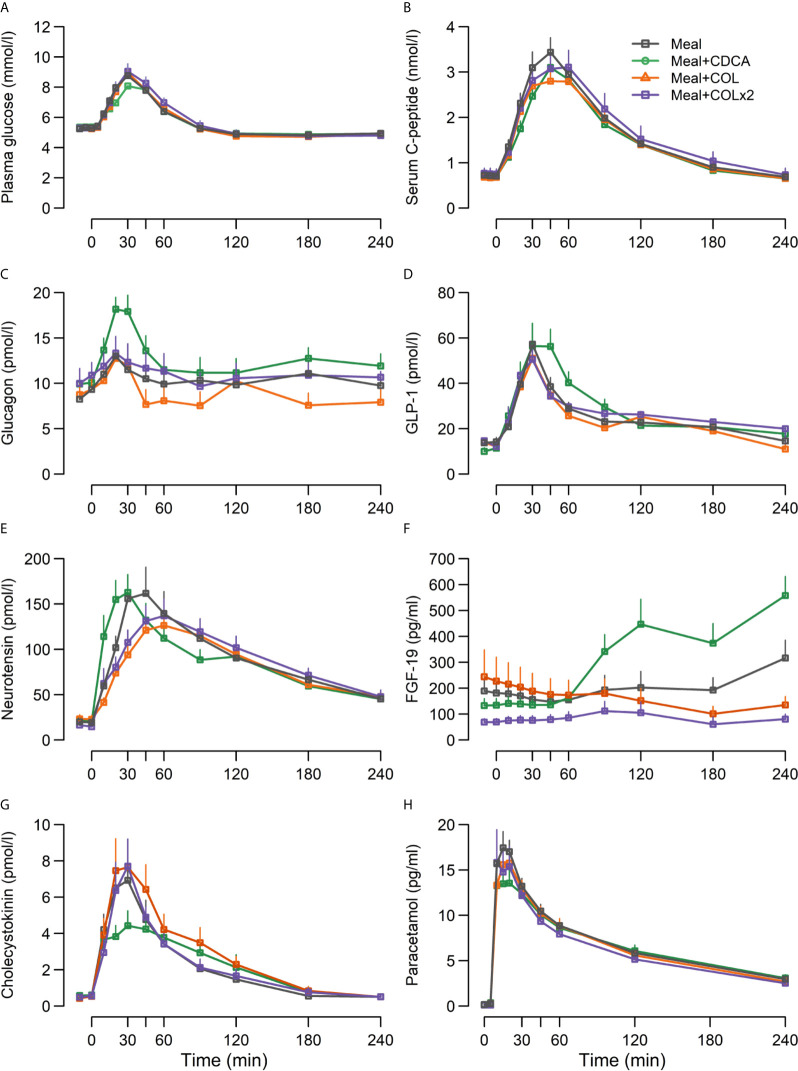
Concentrations of **(A)** plasma glucose, **(B)** serum C-peptide, **(C)** glucagon, **(D)** GLP-1, **(E)** neurotensin, **(F)** FGF-19, **(G)** CCK and **(H)** paracetamol following ingestion of a mixed meal alone (Meal), mixed meal in combination with CDCA with the meal (Meal + CDCA), mixed meal in combination with COL added to the meal (Meal + COL), or mixed meal in combination with COL administered both the night before the study day as tablets and added to the meal (Meal + COL × 2), n = 12. Data are mean ± SEM.

Postprandial GLP-1 responses were prolonged and higher during meal + CDCA compared with the meal alone (*p* = 0.04 for piAUC), whereas peak GLP-1 concentrations were comparable (*p* = 0.3). In contrast, GLP-1 responses after COL administration (both piAUC and peak) were similar to meal alone regardless of COL doses. Concentrations of neurotensin did not differ between test days. Similarly, postprandial CCK secretion appeared unaffected by the addition of CDCA or COL to the meal (**Figure 3**, **Table 4**).

#### Plasma Glucose, Serum C-Peptide, Glucagon, FGF-19 and Paracetamol

Fasting plasma glucose, insulin, and HOMA-IR did not differ between any of the study days (**Figure 3**, **Table 4**).

Adding CDCA to the meal tended to decrease piAUC of plasma glucose concentrations compared with meal alone (*p* = 0.07) and lowered peak of plasma glucose concentrations by ~0.5 mM (*p* = 0.03). In contrast, adding COL to the meal did not change plasma glucose, neither with single nor double COL dosing. Time to nadir and nadir of plasma glucose did not differ between study days.

The piAUC of C-peptide concentrations decreased slightly (7%) during meal + CDCA compared with meal alone (*p* = 0.04), whereas neither doses of COL changed C-peptide concentrations. Peak concentrations of glucagon increased when CDCA was added to the meal and were unaffected by COL.

FGF-19 concentrations increased during meal + CDCA compared with meal alone (*p* < 0.01 for both piAUC and peak) and tended to decrease during COL administration (ANOVA p = 0.14 and 0.12 for peak and piAUC, respectively) (**Figure 3**, **Table 4**).

Time to peak of PCM increased numerically when CDCA was added to the meal and time to peak was unaffected by COL (**Figure 3**, **Table 4**).

#### Measures of Beta-Cell Function

IGI_C-peptide_ was unaffected both by adding CDCA and by both dosages of COL. The beta-cell index covering the whole postprandial period decreased slightly during CDCA and was unaffected by COL (**Table 4**).

## Discussion

We investigated the effect of exogenous CDCA and the importance of endogenous bile acids for postprandial plasma GLP-1 concentrations and for plasma glucose, insulin, glucagon, CCK, neurotensin, and FGF-19 concentrations in individuals that had undergone RYGB by acute administration of CDCA and the bile acid sequestrant COL.

First, we administered CDCA alone or together with COL to RYGB operated participants in the absence of nutrients. This confirmed that CDCA elicits a rise in plasma GLP-1, glucagon, neurotensin, cholecystokinin, and FGF-19 concentrations. This was reported previously both in RYGB-operated (38) and in non-operated subjects after either gastric (15–17) or rectal administration (12, 13). The combined administration of CDCA and COL attenuated both plasma TBA, GLP-1, glucagon, neurotensin, C-peptide, and FGF-19 responses as compared with CDCA alone. Since bile acids stimulate GLP-1 secretion *via* activation of basolaterally located TGR-5 receptors on the L-cell (27, 28), absorption of bile acids across the gut epithelium is required for stimulation of GLP-1 secretion (11). Accordingly, the attenuated bile acid absorption and GLP-1 secretion after combined administration of CDCA and COL in study 1 is consistent with this notion and confirms findings from un-operated subjects (15, 39). Importantly, these results also demonstrate that bile acid sequestrants provide a suitable experimental tool for investigations of the physiological effects of meal-induced bile acid absorption also in RYGB-operated patients. Interestingly, the rise in glucagon and neurotensin concentrations seen during CDCA was also attenuated after administration of COL. The responses of neurotensin to CDCA likely involve similar mechanisms as for GLP-1 (27), whereas stimulation of glucagon secretion by CDCA probably involves other mechanisms. The presence of TGR-5 receptors on pancreatic beta and alpha cells is debated, and the stimulatory effects of glucagon by CDCA may be either indirectly or of intestinal origin (27, 40, 41). The CDCA induced glucagon response could be specific to RYGB given the well documented postprandial hyperglucagonemia due to *de novo* synthesis in the small intestine (26, 40).

Of note, the magnitude of the GLP-1 response obtained after CDCA in study 1 and our previous study (38) was accompanied by a small albeit significant increase in C-peptide without changes in glucose concentrations. When plasma glucose concentrations are within the fasting range, the potentiating effect of GLP-1 on insulin secretion is limited *per se* (42, 43), and therefore the insulinotropic effect of CDCA mediated GLP-1 secretion was expected to be small.

After RYGB, the postprandial GLP-1 secretion is many-fold increased and the contribution of bile acids to this response has been debated but not directly examined previously. Therefore, in study 2 we investigated postprandial GLP-1 concentrations after addition of an exogenous bile acid (CDCA) to a meal or after inhibiting endogenous bile acid reabsorption with COL in RYGB operated subjects. We found that CDCA increased both postprandial TBA concentrations and stimulated overall GLP-1 secretion, indicating that the GLP-1 secreting L-cells were not maximally stimulated by the meal ingestion alone. Also, peak glucagon concentrations were higher during CDCA administration. Meanwhile, both plasma glucose and C-peptide responses were slightly decreased, and no beneficial effect on beta-cell function was found despite the increased GLP-1 secretion. This finding is surprising, but may be explained by lower peak concentrations of plasma glucose after CDCA, which might be mediated by effects of CDCA on intestinal nutrient entry or glucose absorption although time to peak of paracetamol concentrations, reflecting rate of nutrient absorption, was only numerically increased after CDCA. Notable, bile acids have been suggested to be involved in the pathogenesis of postprandial hypoglycemia *via* the bile acids-GLP-1-insulin-axis or indirectly *via* stimulation of FGF-19. Subjects developing postprandial hypoglycemia have been shown to have higher postprandial bile acids levels coinciding with augmented GLP-1 and insulin responses during a mixed meal (44). However, the present study does not support a role for acutely altered intestinal bile acids concentrations for the development of postprandial hypoglycemia as no differences in nadir or in time to nadir of plasma glucose concentrations were found between the four days with mixed meal and differences in plasma bile acid concentrations.

Administration of COL together with the meal decreased postprandial plasma concentrations of TBA with ~68%, indicative of reduced postprandial bile acid absorption, as intended. We added COL in two dosage regimens based on the recent findings with altered bile acid circulation (33), and the extra dose of COL was added to secure binding of the bile acids within the gut lumen already before food ingestion. However, the results obtained from the two different dosage regimens of COL were comparable including fasting bile acid concentrations, which suggests that insufficient inhibition of absorption is not a concern with the single dose of COL. The large decrease in TBA concentrations after COL administration did not affect GLP-1 secretion, which suggests that endogenous bile acids do not appear to be quantitatively important for the total postprandial GLP-1 response after RYGB despite the fact that exogenous CDCA can increase plasma GLP-1 concentrations after RYGB. This difference might be driven by differences in plasma TBA concentrations, which were substantially (nine-fold) higher after adding CDCA suggesting that the levels of endogenous BAs stimulated by meal intake did not have a similar stimulatory capacity. Moreover, CDCA is known to be a relatively good agonist for TGR-5, which is not the case for all endogenous bile acids (27). In previous studies the composition of the different bile acids was not changed after surgery (24, 25) contradicting any special impact of CDCA or other subfractions for the metabolic improvements after RYGB.

The immediate effects of the acute interventions in our studies were anticipated to affect glucose metabolism and GLP-1 secretion mainly through TGR-5 activation. However, bile acids may also affect these axes *via* the FXR pathway although the precise contribution remains controversial and is unlikely to contribute to postprandial responses within the first hour after meal intake, since FXR is a nuclear receptor and responses depend on transcriptional changes (45). FXR activation is supposed to stimulate release of FGF-19, suppress *de-novo* bile acid synthesis, stimulate glycogen synthesis, reduce hepatic gluconeogenesis, inhibit gall bladder emptying, and may also regulate insulin sensitivity (46, 47). In both our studies we observed an increase in FGF-19 concentrations with peak values occurring after the 120 min postprandial timepoint in line with previous studies (25, 48). The observed increase in FGF19 was greatly enhanced by adding CDCA to the meal confirming a link between CDCA and activation of the FXR pathway (49). GLP-1 concentrations peak within the first postprandial hour after RYGB, why any acute effects of bile acids on postprandial GLP-1 and insulin secretion seem independent of FXR activation. This is consistent with findings in some rodent models where activation of the FXR-pathway does not stimulate GLP-1 secretion and may even decrease secretion (18, 27). However, results from other pre-clinical models have opposing findings with increased GLP-1 secretion (50, 51). In addition, targeted knockout of the FXR receptors in mice eliminated the beneficial effects of sleeve gastrectomy on glucose metabolism (52), but this finding can be unrelated to GLP-1 secretion and may also be procedure or species specific. We observed a tendency of decreased FGF-19 concentrations during both doses of COL and GLP-1 secretion was unchanged on these days as mentioned above. In general, the role of FXR activation after bariatric surgery in humans remains unknown and further studies are needed to determine the contribution of FXR activity for the changed glucose metabolism, gut hormone secretion and insulin sensitivity after surgery. With a more chronic treatment with bile acid sequestrants, results might differ with accumulated effects on glucose metabolism and gut hormone secretion by manipulating both TGR5 and FXR activation with more efficient binding of bile acids. Especially, effects from changed FXR activation would be expected to be more pronounced in line with findings in un-operated subjects with type 2 diabetes (53).

The relation between bile acids and CCK in RYGB operated subjects appears to be complex. In unoperated individuals the effect of CDCA (15, 16) and bile acids (54) on CCK secretion was inconsistent, whereas liquid mixed meal tests after adding bile acid sequestrants were associated with increased CCK secretion (53, 55). In the meal study, CCK responses were unaltered by both CDCA and colesevelam. The discrepancy between findings in RYGB-operated and unoperated individuals might involve the apparent dissociation of the normal physiological CCK-mediated regulation of gallbladder emptying and postprandial bile acid concentrations after RYGB, where a larger pool of the bile acids is located within the small intestine in the fasting state, attenuating the importance of postprandial gallbladder emptying (33).

Overall, our findings do not support a major role for endogenous bile acids for the altered postprandial secretions of gastrointestinal hormones or glucose metabolism in RYGB operated subjects. In the present studies, we were unable to identify changes in GLP-1 or measures of beta-cell function after acute attenuation of postprandial bile acid absorption by the bile acid sequestrant COL, although the conclusions based on these finding may be limited by the acute dosage regimen and the inclusion of subjects without type 2 diabetes. Our study also seems to contrast data suggesting that the FXR pathway is crucial for glucose tolerance after bariatric surgery (52), although studies with longer and more chronic administration of sequestrants are probably needed to establish this.

In conclusion, administration of the bile acid sequestrant COL to RYGB operated subjects effectively reduced CDCA-induced circulating bile acids and GLP-1. Further, CDCA administered during meal intake enhanced GLP-1 secretion and decreased both peak glucose and C-peptide concentrations with unaltered beta-cell function. COL added to a mixed meal effectively blocked the absorption of bile acids but neither affected GLP-1, glucose concentrations nor beta cell function, suggesting a limited role for endogenous bile acids in the acute regulation of postprandial glucose metabolism after RYGB.

## Data Availability Statement

The original contributions presented in the study are included in the article. Further inquiries can be directed to the corresponding authors.

## Ethics Statement

The studies involving human participants were reviewed and approved by the Ethical Committee of the Capital Region of Denmark. The patients/participants provided their written informed consent to participate in this study.

## Author Contributions

IJ: Investigation, formal analysis, and writing—original draft. KB-M: Conceptualization, methodology, and writing—review and editing. VK: Resources and writing—review and editing. SV: Resources and writing—review and editing. NW: Resources and writing—review and editing. TC: Resources and writing—review and editing. RK: Resources and writing—review and editing. JR: Resources and writing—review and editing. JH: Conceptualization, methodology, writing—review and editing, and funding acquisition. SM: Conceptualization, methodology, writing—review and editing, and supervision. MS: Conceptualization, methodology, formal analysis, writing—original draft, and visualization. All authors contributed to the article and approved the submitted version.

## Funding

This project has received funding from the European Research Council (ERC) under the European Union’s Horizon 2020 research and innovation program (grant agreement No 695069-BYPASSWITHOUTSURGERY) and from Amager and Hvidovre Hospital Research Foundation.

## Conflict of Interest

RK works for Novo Nordisk A/S, but was at the time of his contribution exclusively employed by University of Copenhagen. TC works for Novo Nordisk A/S and own shares in Novo Nordisk A/S and Zealand Pharma A/S. All other authors declare no conflict of interest in relation to the current manuscript.

## Abbreviations

BA, Bile acids; CCK, Cholecystokinin; CDCA, Chenodeoxycholic acid; COL, Colesevelam; FGF-19, Fibroblast growth factor 19; GLP-1, Glucagon-like peptide-1; piAUC, positive incremental area-under-the-curve; RYGB, Roux-en-Y gastric bypass; TBA, Total bile acids; VAS, Visual analogue scale.
